# Osteoporosis and risk of fracture: reference class problems are real

**DOI:** 10.1007/s11017-022-09590-3

**Published:** 2022-09-17

**Authors:** Nicholas Binney

**Affiliations:** Department of Medical Ethics, Philosophy and History of Medicine, Room Na 24-15, PO Box 2040, 3000 CA Erasmus, Rotterdam, MC The Netherlands

**Keywords:** Reference classes, Osteoporosis, Disease concepts, Boorse, Kingma, Risk of fracture

## Abstract

Elselijn Kingma argues that Christopher Boorse’s biostatistical theory does not show how the reference classes it uses—namely, age groups of a sex of a species—are objective and naturalistic. Boorse has replied that this objection is of no concern, because there are no examples of clinicians’ choosing to use reference classes other than the ones he suggests. Boorse argues that clinicians use the reference classes they do because these reflect the natural classes of organisms to which their patients belong. Drawing on a thorough exploration of how the disease osteoporosis is defined in adults, I argue that clinicians do indeed make choices about which reference classes to use in diagnosis. Clinicians use young adult reference classes to diagnose osteoporosis in elderly patients. They also use young female reference classes to diagnose osteoporosis in elderly males. Clinicians adjust their reference classes so that the diagnosis of osteoporosis reflects a person’s risk of sustaining a fragility fracture. The ethical intuition that people with the same risk of fracture should receive the same diagnosis overwhelms the naturalistic intuition that reference classes should reflect natural classes of organisms of uniform functional design. Clinicians construct a variety of reference class types, including pathological reference classes and epidemiological population-specific reference classes, to serve this ethical intuition. I show how clinicians use several reference classes at once so that they can more accurately predict risk of fracture. Ultimately, the reference classes chosen and used in medical practice are quite different from those proposed in naturalistic philosophy of medicine.

## Introduction

Reference classes are central to Christopher Boorse’s biostatistical theory of health and disease (BST). According to the BST, diseases are atypically diminished functioning of an individual’s biological parts or process [1, 2]. The BST defines biological function as the typical contribution of a part or process to an individual’s survival and/or reproduction. The BST requires reference classes to establish both what the functions of an individual’s parts and processes are, and how efficiently an individual’s parts and processes are functioning, in order to define disease. In this paper, I focus primarily on this latter use of reference classes—determining level of functioning—arguing that the BST’s account of reference classes does not conform to medical usage regarding the disease osteoporosis.

In the BST, an individual’s level of functioning is compared to the average for that individual’s reference class. Whether one’s level of functioning is above or below this average depends on the level of functioning of the other members of the reference class. Thus, altering the reference class to which one belongs has the power to alter one’s level of functional efficiency within the BST, and thus to alter one’s disease status.

According to the BST, reference classes are age groups, of a given sex, of a given species [1, 2]. So, any individual’s reference class consists of other individuals of the same age, sex, and species.^2^ Boorse argues that these reference classes are each a “natural class of organisms of uniform functional design” [1, 2]. He argues that clinicians^3^ use these concepts when they define the reference classes that they use to define diseases. So Boorse argues that clinicians use age groups, of a sex, of a species as references classes to define diseases, and that clinicians do so because these groups are natural classes of organisms with uniform functional design.

Several other philosophers, including Elselijn Kingma [3], have argued that clinicians may use different references classes to define disease [4, 5]. By doing so, clinicians would be adopting different “candidate concepts” of disease to that described by the BST. Boorse denies that this is the case, arguing that the alleged problem of having many reference classes to choose from, each producing its own concept of disease, is a fiction dreamed up by armchair philosophers: “I try to choose that analysis which best fits medical usage. The medical concept of health that I seek to analyze already exists as a target. “Candidate concepts,” by contrast, exist only in the minds of philosophers” [2].

Boorse also argues that the science of physiology is used by clinicians to define diseases, as it is used to define biological function. Boorse argues that epidemiology is not used by clinicians to define diseases, as “epidemiology seems to presuppose a concept of disease, not to define one” [2]. In contrast to this, Élodie Giroux has argued that medicine’s use of epidemiology and risk factors introduces a variety of different reference classes into medical practice, generating the multiple candidate concepts that Boorse denies exist [6]. Epidemiology studies populations of humans, not the whole human species at once, making risk factors relative to these populations. These populations, then, could be understood as reference classes. Giroux argues that both epidemiology and physiology are used to define disease [6, 7]. Boorse rejects this, arguing that epidemiology does not itself define disease, and that epidemiologists always define disease in the same way in all populations: “I am not sure that epidemiologists ever claim that the same condition (e.g., diastolic blood pressure over 90) is a disease in one group but not in another group. Although they work with specific populations, that does not mean that they are trying to define, say, ‘disease of a Framingham male’” [2, p. 703].^4^ Boorse thus denies that epidemiology influences how disease is defined in medicine, and that it does not produce a variety of different candidate concepts for clinicians from which to choose.

Despite these denials, Boorse does recognize that the disease *osteoporosis* presents a problem for his theory. As he notes, the reference class used to determine whether elderly women have osteoporosis is not other elderly women, but young women: “For example, current views of osteoporosis precisely judge older women by young ones, defining it as a bone mineral density (BMD) more than 2.5 standard deviations below the mean for young, healthy adults” [2, p. 721]. In current medical practice, bone mineral density relative to a *young* female reference class indicates disease in *elderly* women. This practice presents a problem for Boorse’s theory because it is an example of clinicians using what is (according to his theory) the wrong reference class.

Boorse argues that this is not necessarily catastrophic for the BST. He uses age to define reference classes because he argues that humans, like other creatures, have life stages through which they develop, changing how they function as they do. Consequently, Boorse argues that male and female humans need to be divided into age groups to reveal the natural classes of organism to which they belong. However, Boorse entertains the possibility that old age is not a proper life stage for humans, arguing that biologists have not settled the matter: “And, in general, it seems to be a live scientific issue whether senescence is a selected, genetically controlled life stage in the human design. Some, therefore, might argue that whether ‘normal aging’ is pathological is an empirical question” [2, p. 721]. If young adulthood is the last life stage of the human, then there may be a naturalistic justification for using the young adult reference class to define disease in older adults. Boorse recognizes the problems this move may cause, as it would make age-related changes in function, such as the menopause, diseases, observing that “medicine does not seem ready to call menopause pathological, as the revision makes it” [2, p. 271]. Nevertheless, debate within medicine about the appropriate reference classes to use is not catastrophic to the BST, so long as this debate is about which reference classes are the correct natural classes of organism.

Whether debates in medicine about which reference class to use are reducible to debates about biological reality can be explored empirically. The medical literature on osteoporosis is indeed a good place to explore such debates, especially in relation to the role reference classes and epidemiology may play in generating different candidate concepts of disease.

Following a short introduction to osteoporosis in the next section, I conduct a thorough analysis of how and why reference classes are used to diagnose osteoporosis. Clinicians use a young female reference class to diagnose osteoporosis in older women by generating what is known as a T-score. The reason elderly women are evaluated against young women is to allow the risk of fracture to be evaluated. Indeed, the desire to predict and compare risks of fracture leads clinicians to use the young *female* reference class to define disease in older *men*, ignoring not just age but sex as well. As surprising as these choices of reference class might be, they still define health as being typical of a *normal* population. Exploring osteoporosis shows a medical interest in defining health as being atypical for a *pathological* population. Furthermore, bone mineral density is an imperfect tool for the evaluation of fracture risk, as many patients with normal bone mineral density will sustain fractures, and many people with low bone mineral density will not. Consequently, many clinicians have tried to develop tools that can be used to predict fractures more accurately by incorporating additional risk factors into models of fracture risk. I close by discussing how many of these ideas are combined in tools designed to evaluate risk of fracture more accurately than bone mineral density alone, such as FRAX. Such tools reveal the central role that epidemiology plays in defining reference classes and different concepts of disease.

A thorough exploration of the use of reference classes to diagnose osteoporosis shows that clinicians are not trying to compare patients to the correct natural class of organisms. They are doing something much more pragmatic—trying to determine a patient’s risk of sustaining a fragility fracture. They use a wide variety of reference classes and disease concepts to achieve this pragmatic goal.

## Introduction to osteoporosis

A fragility fracture is a broken bone caused by a low level of physical trauma. One in three women over fifty years old will sustain a fragility fracture, as will one in five men [11]. In the European Union, 3.5 million people sustained fragility fractures in 2010 [11]. In the UK, there are over half a million fragility fractures suffered annually, most often in elderly people [12]. Common sites for fragility fractures include the vertebrae, the hip, the forearm and humerus, and such fractures are associated with significant morbidity and mortality. “Approximately 53% of patients suffering a hip fracture can no longer live independently and 28.7% die within 12 months of the fracture. Only 54% of individuals admitted from home with a hip fracture return there within 30 days” [12]. Across the European Union, there were forty-three thousand fracture related deaths in 2010, around three-quarters of which were related either to a hip or vertebral fracture [11].

Age related weakening of the skeleton is associated with the disease osteoporosis. Osteoporosis literally means ‘bones with little holes’ or ‘porous bones’ [13, 14]. The term has been in use since the 1820s in relation to the reduction in the quantity of bone seen in people as they age. By the mid-twentieth century, clinicians had recognized that most people lost a significant amount of bone mass as they aged [13]. Osteoporosis was connected with post-menopausal oestrogen loss in the 1940s, and it was differentiated (by some) from other bone diseases such as osteomalacia and osteogenesis imperfect [14].

Until very recently, osteoporosis lacked a precise and standardized definition. Some authors defined osteoporosis as a disease of post-menopausal women, whereas others defined it as a disease that could affect men as well. Some defined it as a disease of the aged, others allowed younger people to have the disease as well. Some defined it as a reduction of bone strength, whereas others defined it as a change in bone quality involving the loss of mineral and protein content. Some defined it as a clinical syndrome involving pain, collapse of the patient’s spine, and fragility fractures, instead of focusing on the disease’s aetiology [14]. Often, these definitions did not determine how the diagnosis should actually be made, leading to variation in clinical practice [14]. Throughout the twentieth century, X-rays were used to detect fractures, collapsed vertebrae and to assess the radiodensity of bone, but this only permitted diagnosis following the development of severe pathology [14]. This led to attempts to develop a method to assess the mineral content of bone, or bone density, that could help identify patients with fragile skeleton before they sustained a fracture.

In the 1980s dual-energy X-ray absorptiometry (DXA) was developed to provide reliable, safe and relatively inexpensive bone mineral density measurements. Reduced bone mineral density is correlated with bone strength and fracture risk. The ability to measure bone mineral density prompted practitioners to redefine osteoporosis in terms of these measurements. “As quantitative bone density measurements replaced qualitative X-ray and biopsy examinations, definitions and diagnostic methods began to shape each other” [14]. Translating this medical discussion into the terms of the BST, clinicians now had a continuous parameter that could serve as a ‘function’ of bone. In order to use this measurement to diagnose disease, however, they needed a standard by which to judge the adequacy of a patient’s bone density. Clinicians felt that “the main problem is to define the standard against which this reduction should be measured” [15] and “As long as the standard is not expressly specified, the definition remains invalid” [16]. Medical researchers needed a reference class.

A series of international conferences held by European and American osteoporosis associations around 1990 failed to produce an agreed upon definition [14]. However, a conference sponsored by the World Health Organization (the WHO) in 1992 did manage to produce consensus. The WHO’s report (1994) [17] discussed the various definitions proposed at this conference. The WHO defined normal bone density, low bone density (osteopenia), and bone density so low as to warrant the diagnosis of osteoporosis. The WHO (2007) also defined severe osteoporosis in patients with low bone mineral density and fragility fractures. These definitions were offered in terms of a patient’s ‘T-Score’: the number of standard deviations an individual fell below the young adult mean [18].

- *Normal.* A value for BMD [bone mineral density] or bone mineral content (BMC) within 1 SD of the young adult reference mean.
- *Low bone mass (osteopenia).* A value for BMD or BMC more than 1 SD below the young adult mean but less than 2.5 SD below this value.
- *Osteoporosis.* A value for BMD or BMC 2.5 SD or more below the young adult mean.
- *Severe osteoporosis (established osteoporosis).* A value for BMD or BMC more than 2.5 SD below the young adult mean in the presence of one or more fragility fractures [17].

As discussed in the philosophical literature, it is notable that this definition uses *young adult* females as a reference class for *older*, post-menopausal women. As bone mineral density typically falls as people age, this way of defining disease results in large numbers of people being diagnosed with a disease. In 2010, twenty-two million women and five-and-a-half million men were thought to have osteoporosis, using this definition [11]. Although bone mineral density is correlated with bone strength and fracture risk, a substantial proportion of people with low bone mineral density will never sustain a fracture, and a substantial proportion of people with more normal bone mineral density will sustain a fragility fracture. Consequently, many people diagnosed with osteoporosis will not go on to sustain a fracture.

Some clinicians have argued that the WHO’s definition of osteoporosis captures too many people. “In 1994 a small study group associated with the World Health Organization defined “normal” bone mineral density as that of young adult women, instantly categorizing many older women as having abnormal bones” [19]. In parallel with the problems associated with other diseases defined using young adult reference classes, such as chronic kidney disease [20–22], some clinicians have argued that this is a foolish way to define disease. Such clinicians are worried that this definition casts too wide a net, capturing too many people who do not benefit from the diagnosis, and leading to overdiagnosis. Others disagree, arguing that ageing should be understood as a disease [23, 24]. This approach to defining disease is medically controversial, but the focus of this paper is not to discuss whether it constitutes overdiagnosis, or whether it is the correct way to define the disease. Rather, my goal is to explore how and why reference classes are used to define osteoporosis. According to the BST, medical selection of reference classes should reflect attempts to use the appropriate natural class of organisms. Exploring the medical literature on osteoporosis permits investigation of whether this is indeed the case.

## The young reference class: the T-score

Exploring how using young adult reference classes to generate ‘T-scores’ reveals that intuitions about sex differences and natural ageing are important to the diagnosis of osteoporosis. However, the desire to predict fragility fractures is even more important, and overwhelms these naturalistic intuitions.

### The use of a young female reference class to evaluate older women

When discussing the appropriate reference class to use when diagnosing osteoporosis in older women, some clinicians do make arguments that have a Boorsian flavor to them. That bone mass falls as people age has been known for decades. Research into the history of osteoporosis shows that the universal nature of low bone density in the elderly has presented a conceptual problem for clinicians [13]. If all people lose bone mass as they age, at what point should such losses be considered disease? Indeed, why should such losses be seen as disease at all? For example, Christopher Nordin (an endocrinologist who played a major role in osteoporosis research in the twentieth century) worried that the incidence of osteoporosis “is known to rise with age, particularly in women, but it is far from clear whether it is a ‘normal’ accompaniment of ageing” [25]. The concept of a universal disease made many clinicians feel uneasy: “The concept that all women and most men become osteoporotic, if they live long enough, is distasteful to some” [15]. To get past this problem, Nordin cited precedent from other areas of clinical physiology as a justification for using a young reference class, saying that “For this there are ample precedents in other fields of clinical physiology where the normal range is usually derived from young healthy adults. The same standard should be applied to bone” [15].

The intuition that age-adjusted reference classes should always be used is shared by many clinicians concerned about overdiagnosis [19], and indeed by members of the public. In a focus group run for the public on osteoporosis “participants expressed unease that “normal” was not adjusted for age, a sense that the definition was “strange” and nonsensical, and a view from some that it should be reformed” [26]. “Our findings have identified a potential gap between community expectations and the way some diseases are constructed and thresholds set, unadjusted for age and axiomatically causing overdiagnosis” [26]. Although such conversations may be held up as evidence that people do worry about whether their reference classes reflect what is normal, and perhaps natural, a different concern dominates the medical literature on osteoporosis—the risk of sustaining a fragility fracture.

Traditionally, many clinicians had reserved the diagnosis of osteoporosis for those patients with severe skeletal deformity and fragility fractures. Once bone density measurement became available, however, clinicians like Nordin were keen to distinguish fragility fractures from osteoporosis as a disease process, tightly linking the disease with fracture risk. “What osteoporosis does is to increase the fracture risk, not cause the fracture” [15]. Similarly, the WHO’s (1994) physiological definition of osteoporosis defines the disease as the weakening of bone increasing fracture risk: “A disease characterized by low bone mass and microarchitectural deterioration of bone tissue, leading to enhanced bone fragility and a consequent increase of fracture risk” [17]. The reason clinicians were interested in measuring bone density is because this might allow them to identify those people whose skeletons were becoming weakened, in order to predict who would sustain a fracture.

Bone mass decreases at most skeletal sites with age, and there is a high correlation between bone mass and bone strength tested in vitro. In addition, measurement of bone mass allows the assessment of fracture risk. Risk of fracture increases continuously as bone mass declines. [17]

The desire to predict fragility fractures provides an explanation for the attitude that statistically normal loss of bone during ageing should be a disease. Clinicians felt compelled to recognize normal age-related decreases in bone strength as a disease because of the resulting fragility fractures, *despite* the conceptual problems this caused.

The role that a desire to predict fractures played in choosing a reference class is made explicit in the medical literature on osteoporosis. The use of age-adjusted reference classes was rejected by many precisely because risk of fracture rises significantly with age, and high risks of fracture are quite normal in elderly populations: “This is due to the fact that bone density ordinarily decreases with age. Thus, the use of an age-adjusted normal range does not provide a reliable means to accurately identify patients who are at risk for fracture-the major goal of spinal bone density measurements” [27; see also 28]. The ability to assess fracture risk is seen as the main selling point of the use of the T-score, which uses the young adult population as a reference class for older patients. “As T-scores decrease, the relative risk for fracture increases. This principle makes the T-score an effective means of identifying those individuals at increased fracture risk and offers a cut point that allows for a diagnosis of osteoporosis” [27]. Rather than focusing on finding the most natural reference class, clinicians discussing osteoporosis focus on predicting an adverse clinical event: the fragility fracture. The desire to predict an adverse clinical event has overwhelmed the intuition that statistically normal age-related changes should not count as diseases.

### The use of young female reference class to evaluate older men

Perhaps counterintuitively, for over twenty-five years the WHO (1994) [17] has defined osteoporosis in older women using young women as a reference class. Since then, the definition of osteoporosis has become more counterintuitive still. In addition to defining osteoporosis in older women using a young female reference class, the WHO (2007) [18] have also defined osteoporosis in *older men* using a young female reference class.

The validation of BMD measurements and the increase in epidemiological information permit diagnostic criteria for osteoporosis to be more precisely defined than previously. The international reference standard for the description of osteoporosis in postmenopausal women and in men aged 50 years or more is a femoral neck BMD of 2.5 SD or more below the young female adult mean, using normative data from the NHANES reference database on Caucasian women aged 20–29 years. [18]

NHANES is the National Health and Nutrition Examination Survey, a survey designed to assess health and nutritional status in adults and children in the US. This American reference population is supposed to be used to calculate T-scores for patients anywhere in the world [28]. So, the diagnosis of osteoporosis in an elderly non-Caucasian man is based on a comparison of that man’s bone mineral density with the average value for Caucasian women in their twenties. This position is not only advocated by the WHO, but also by the International Osteoporosis Foundation, and the International Society for Clinical Densitometry [28, 29]. From the point of view of the BST, this reference class does not just use the wrong age, but the wrong sex and the wrong race. Only the species is in agreement with the BST.

This recommendation “is understandably controversial” [29]. “Until recently, separate gender-specific databases were used to derive the T-score” [29]. Such comments indicate that, again, clinicians do have intuitions that direct them to use sex-specific reference classes. Given this, it is again reasonable to wonder about what it is that has over-ridden these intuitions; and again, the over-riding factor is the risk of fracture.

As discussed, risk of fracture is related to bone mineral density (BMD), as measured by DXA. Density is usually understood as the mass of something divided by its *volume.* Bone density as measured by DXA, however, measures the mass of bone in the *area* assessed. DXA measures mass per unit area (grams/cm^2^), not mass per unit volume (grams/cm^3^). Men often have a slightly lower volumetric density of bone than women, but men also often have much larger bones than women do. So, even though the volumetric density of bone is not greatly different between men and women, men (on average) have much higher peak bone density as measured by DXA. “Peak bone mass as measured by DXA is greater in men than women, because of larger bone size in men and the fact that the 2-dimensional depiction of BMD (g/cm^2^) by DXA is heavily influenced by bone size” [29]. Although men, on average, do not have greater volumetric bone density than women, they do have much greater bone mass.

All things being equal, a greater amount of bone provides a greater amount of strength. As a result, men’s bones are often stronger than those of women, and have a lower risk of fracture. Consequently, to reach the same risk of fracture men often must lose more bone than women do [29]. A man’s BMD needs, on average, to fall further than a women’s to reach the same low level of BMD. As it happens, men and women with the same BMD have approximately the same risk of fracture: “In summary, it appears that at the same DXA-measured BMD, men and women are at approximately the same fracture risk” [29]. So, a man’s BMD has to fall much farther from their sex’s average value to reach a certain risk of fracture than does a woman’s. When fracture risk is at the same high level, men are much more atypical than women.

As discussed, BMD and fracture risk are evaluated using the T-score. This represents the number of standard deviations an individual’s BMD falls below the average of a reference class. As the average BMD for men is higher than for women, a man with a low BMD is much further away from their sex’s average value than is a woman with the same BMD. A man’s T-score will therefore be lower (more negative) than a woman’s T-score with the same BMD, if sex-specific reference classes are used. Wherever the line is drawn to set the T-score that distinguishes osteoporosis from osteopenia, men will cross this line at lower risk of fracturing than women if a sex-specific reference class is used.

Now, clinicians might just accept this, if they accept that tracking statistical normality is what is important to defining disease. However, in the osteoporosis literature, clinicians do not do this. As men and women with the same BMD have the same risk of fracture, many clinicians feel that it is unreasonable to diagnose disease in some people but not in others with the same level of risk. Consequently, these clinicians feel that the *same* reference population should be used to judge the bone mineral density of any elderly person, be they male or female, to prevent variations in T-score where there is no variation in risk of fracture: “In summary, it appears that at the same DXA-measured BMD, men and women are at approximately the same fracture risk. As such, use of the same database to derive the T-score is reasonable” [29].

Despite considerable differences in average peak bone mass, areal bone density and bone strength between men and women, clinicians choose not to use sex-specific reference classes to diagnose osteoporosis. They judge the BMD of old men against a young female reference class because this helps them to track an individual’s risk of fracture, even though this inhibits their ability to determine whether that individual is typical for a class of organisms of similar functional design.

Given that risk prediction appears to be the aim of these clinicians, one might wonder why they don’t just report a patient’s bone mineral density with the associated risk of fracture directly, instead of worrying about whether this is typical for any reference class. Why bother with reference classes at all? An answer to this question is provided in the literature on osteoporosis. Several different manufacturers make the machines that measure bone density. For technical reasons, these different machines do not give the same results when used on the same patient, making their results incomparable [29, 30]. However, these results can be converted to a T-score. This makes the results comparable, as the T-score represents the number of standard deviations a machine’s result is below the young average for that machine. This value can be compared with the number of standard deviations that another machine’s result is below the young average for that other machine, even if direct comparison of these machines’ results is meaningless.

If all DXA instruments measured BMD identically, there would be no need for a T-score; unfortunately, this is not the case. To avoid confusion that would result from instrument specific numerical BMD cutpoint values, the T-score concept was suggested whereby each patient’s value is compared with a young normative database generated on the same device. [29]

Such clinicians see the adoption of the T-score by the World Health Organization as a great advance for the study of osteoporosis. The WHO (1994) sought to provide criteria that could be used for epidemiological monitoring of global differences in skeletal fragility, but they also provided diagnostic criteria for osteoporosis with therapeutic intervention in mind [17]. The WHO set the diagnostic threshold for osteoporosis to locate patients at high risk of sustaining fragility fractures in the future: “The implication is that such women should be offered intervention” [17]. Focusing on the risk of fracture, rather than on the presence of fracture, provided a reasonable basis for therapeutic interventions before the development of severe disease, including hormone replacement therapy. The adoption of the T-score allowed the diagnosis of osteoporosis to be *based* on the risk of fracture.

Since fracture is strongly related to reduced bone mass and because risk is related on a continuum to BMD, the level of risk as defined by the T-score was a key moment in the field. While the original WHO classification was intended for a population-based prevalence approach, it led to a diagnostic classification of osteoporosis based on risk of fracture. [29]

Some clinicians see the T-score not as an expression of how far a patient’s bone mineral density deviates from some natural standard, but rather as an expression of the risk of an adverse clinical event.

Exploring how the T-score is used to define osteoporosis has revealed circumstances under which clinicians will make comparisons between people of different ages, and even between people of different sexes. However, even here comparisons are still being made between an individual’s bone mineral density and the average bone mineral density of a *normal* population, representing the whole population. Instead of using a normal population, it is also possible that disease can be defined relative to a *pathological* population.

## The pathological reference class: fracture threshold

In 1994, when the WHO put forward its definition of osteoporosis, they considered several alternative definitions. In addition to the T-score, the WHO also considered defining osteoporosis relative to a “fracture threshold” [17, 31, 32]. In this approach, clinicians look at populations of patients *who have sustained fragility fractures* and use these patients to set level of bone mineral density above which fractures are unlikely to occur. The WHO indicates that this could be done by plotting the distribution of the numbers of patients with fragility fractures according to their bone mineral density, finding the mean value of BMD in this *fracturing population*, and locating the value of bone mineral density that is two standard deviations *above* this mean^5^. Patients with bone mineral density below this value would be diagnosed with osteoporosis, and those above would be considered healthy.

A second approach has been to characterize the osteoporotic population to derive a “fracture threshold” based on the range of bone mineral density measurements in the population with vertebral or hip fractures. This can be arbitrarily set, for example, at 2 standard deviations above the mean value of patients with osteoporotic fracture. [17]

Compared to the T-score, or to how disease is conceptualised in the BST, here everything is back to front. Instead of looking at the *whole* population of patients, comprised largely of people who are *healthy*, in this approach the *diseased* population is examined. Instead of setting the threshold between health and disease at some level *below the healthy* average, the threshold between health and disease is set at some level *above the diseased* average. Here, clinicians are determining what is normal from their knowledge of what is diseased, not the other way around. The use of a *pathological reference class* by these clinicians to determine what is normal employs a very different concept of disease to the one outlined by the BST.^6^

The WHO (1994) [17] did not decide to use the fracture threshold concept to define osteoporosis, but this does not seem to be because they thought it employed the wrong reference class. Instead, they drew attention to empirical results that made the search for a threshold of bone mineral density at which bones became so weak that they would fracture implausible. They pointed out that the probability of fracturing amongst patients with the same bone mineral was much greater amongst older patients than younger ones. They also pointed out that there was a significant overlap in BMD between the populations of patient who would eventually sustain a fragility fracture and those that would not [17]. These findings showed that there was no level of bone mineral density below which all bones would eventually fracture and above which no bones would fracture. This made it difficult to conceive of a true fracture threshold. “There is thus no true ‘fracture threshold’, even though the term is widely and usefully employed to indicate a threshold for intervention” [17].

However, this problem is not unique to the fracture threshold concept. The WHO also argues that the failure of bone mineral density alone to predict who would sustain a fragility fracture was a problem for all the approaches under consideration, including for the T-score [17]. If using either the healthy or pathological reference class could point to an obviously valuable level of bone mineral density then this would count in its favor. Unfortunately, since the pathological (fracturing) and healthy (non-fracturing) populations had overlapping bone mineral densities, neither approach could point to such a level. Consequently, several researchers seem ambivalent about which approach to take, describing the fracture threshold approach as “the most straightforward” [17] and the T-score as a “reasonable alternative” [31]:

Several approaches have been taken to define osteoporosis on the basis of bone mineral measurements. The most straightforward is to define a fracture threshold (even though the gradient of risk is continuous), namely, the cutoff for bone mineral that captures most patients with osteoporotic fractures. This can be variously and arbitrarily set, for example at the mean, or at 1 or 2SD above the mean value of patients with osteoporotic fractures. Alternatively, it can be set somewhere below the mean of the young healthy adult reference range. [32]

Entertaining the possibility of using either a pathological or a young health reference class does not seem to have presented these clinicians with much conceptual difficulty. They act as though all either approach could do was provide a language in which to describe the level of BMD that was “arbitrarily” chosen for other reasons.

To set the diagnostic threshold for osteoporosis, the incidence of disease, treatment options, the goals of testing and the ability of bone mineral density to predict fractures were all taken into account. Thirty to forty% of women would sustain a fragility fracture at some point in their lives, making the lifetime incidence of this problem high [32]. The WHO claimed that, in the 1990s, none of the available treatments for osteoporosis could restore skeletal strength once it had been lost, which they felt “argues for assessment and intervention early in the natural history of the disorder” [17]. The WHO sought to assess selected populations of asymptomatic patients in higher risk groups (such as recently postmenopausal women) to identify smaller groups of asymptomatic women at even higher risk who, as a group, would benefit from treatment^7^. The risk of sustaining a fracture approximately doubled with each standard deviation below the young healthy average bone mineral density, making bone mineral density a useful (although imperfect) tool to assess fracture risk in populations of asymptomatic patients [17]. They sought to set the diagnostic threshold at a level that would capture as many of the people who would fracture as possible without needlessly capturing too many people who would never fracture. They decided on 2.5 SD below the young average bone mineral density as a pragmatic compromise between extreme alternatives. This captured 30% of all post-menopausal women in the diseased group, which was commensurate with the lifetime risk of fracture in postmenopausal women. “Fortuitously” [17], this compromise level was “also close to the imagined fracture threshold, thus satisfying those groups who favored the idea of a fracture threshold” [14]. So long as an appropriate level of bone mineral density was chosen, these clinicians did not seem to mind how this was expressed.

Furthermore, the WHO emphasized that the approach they took to defining osteoporosis should not be thought of as universal. In different epidemiological and clinical contexts, different approaches may be more suitable. Even when considering their approach is context dependent, they only seem concerned about assessing fracture risk, and not about the nature of their reference class.

It is important to recognize that the foregoing considerations may not be applicable worldwide since there is no universally agreed definition of acceptable risk in the community and since the incidence of osteoporotic fracture varies markedly (more than 10-fold in different European countries, for example), which also affects the appropriateness of the cut-off. [17]

The decision to use a young healthy reference class rather than a pathological reference class does not appear to have involved protracted discussions of which reference class captured the correct natural class of organisms. However, it did involve protracted discussions of the level of bone mineral density that would be useful to highlight given this particular epidemiological and clinical context.

Exploring the medical literature on osteoporosis has revealed the use of several different reference classes. However, in each case, only one reference class is used at a time. The desire to more accurately predict risk of fracture has led clinicians to use several different reference classes at once, combining many of the ideas already discussed.

### FRAX

The WHO defines osteoporosis using bone mineral density as expressed as a T-score. The limitations of this approach have always been recognized [17]. Bone is a complex material, and its mechanical properties cannot be reduced to bone mineral density [40]. Most tissues are composed largely of cells surrounded in smaller amount of extracellular matrix. Bone, however, is comprised of a small number of cells set within a much larger volume of extracellular matrix, which is itself a combination of organic and mineral material. Bone is a dynamic tissue, which is constantly being laid down and reabsorbed in different configurations to cope with the changing mechanical stresses imposed upon it [41]. Bones themselves have outer shells of hard or cortical bone, which has a solid structure, surrounding regions of spongy or trabecular bone, which has a honeycomb structure. The mechanical properties of a bone will depend on the quality of its organic matrix, its degree of mineralization, on the thickness of cortical bone and configuration of trabecular bone [39]. Anyone who has put scaffolding together knows that the strength of the structure depends upon the way it is put together and not simply on the amount of material in the scaffold. Consequently, measuring bone mineral density does not tell the whole story of bone strength.

This is reflected in the epidemiology of bone mineral density and fragility fractures. Populations of patients with low bone mineral density have a greater proportion of individuals who sustain fragility fractures than populations with higher bone mineral density do. Overall, the probability of sustaining a fragility fracture falls as bone mineral density rises. Nevertheless, some patients with low bone mineral density will not fracture, and some patients with higher bone mineral density will fracture. Indeed, most fragility fractures will occur in patients with levels of bone mineral density much higher than the threshold for the diagnosis of osteoporosis. Populations of fracturing and non-fracturing patients have levels of bone mineral density that *overlap*, and this overlap is quite large [17, 18, 31, 42].

### Defining osteoporosis in terms of fracture risk

In order to combat this problem, and more accurately distinguish those patients who will fracture from those who will not, clinicians have built models of the risk of fracture using several other factors in addition to bone mineral density. One such model is FRAX, which was developed in the late 2000s by a team in the United Kingdom at a WHO collaborating center. FRAX incorporates information about age, sex, body mass index, history of previous fracture, history of whether a parent had fractured a hip, smoking, alcohol consumption, steroid (glucocorticoid) use, and the presence of rheumatoid arthritis. Each of these factors was found to influence the risk of fracture largely independently of bone mineral density [43]. So older patients are more likely to sustain a fragility fracture, as are patients with a body mass index of 19 kg/m^2^ or lower, as are patients who have previously sustained one, as are patients with a parent who has fractured a hip, and so on. Researchers developing FRAX use country specific epidemiological data to build a tool that uses this information to produce a more accurate estimation a patient’s ten-year risk of fracture than using bone mineral density alone [42].

Arguably, some clinicians have started to conceive of osteoporosis as having a high risk of fracture, rather than as having a low bone mineral density. Consider osteoporosis as diagnosed using the T-score. While osteoporosis has in the past been understood as the fragility fractures themselves, according to the 1994 WHO definition, osteoporosis is not the fractures themselves, but is a deterioration in a set of characteristics of bone that can lead to fractures: “A disease characterized by low bone mass and microarchitectural deterioration of bone tissue, leading to enhanced bone fragility and a consequent increase of fracture risk” [17]. So, the characteristics that define osteoporosis are low bone mass, microarchitectural deterioration of bone tissue, enhanced bone fragility, and increased fracture risk. In this 1994 formulation, bone mass and microarchitecture may have conceptual priority, as they are said to *lead* to fragility and increased risk of fracture. This may imply that these latter characteristics are a *consequence of having* osteoporosis, as opposed to *part of being* osteoporosis. Indeed, the WHO was careful to distinguish osteoporosis itself from the increased risk of fracture: “In consideration of these issues, it is important to distinguish assessments of (fracture) risk from the diagnosis of disease (osteoporosis). Depending on factors such as rates of bone loss, falls and life expectancy, the disease may or may not give rise to symptoms” [17].

It seems, however, that conceptual priority concerning the defining characteristics of osteoporosis has shifted. The WHO (2007) [18] have emphasized that the whole purpose of focusing on areal bone mineral density was to assess the risk of fracture. “The pivotal requirement for the use of bone mineral testing in diagnosis and assessment of osteoporosis is its performance characteristics for fracture prediction” [18]. The implication of this is that bone mineral density measurement is not a direct and perfect measurement of the characteristic that defined osteoporosis. Rather, low bone mineral density as measured by the T-score is just a proxy for osteoporosis, an imperfect indicator of its presence. Here, conceptual priority is given to the fragility of the skeleton, and particularly to the risk of fracture. Such a view of osteoporosis is made explicitly in contemporary medical literature, including in a position statement by the National Bone Health Alliance Working Group in the US [27].

As T-scores decrease, the relative risk for fracture increases. This principle makes the T-score an effective means of identifying those individuals at increased fracture risk and offers a cut point that allows for a diagnosis of osteoporosis. However, it is clear that there are other ways to identify individuals at high fracture risk, including the occurrence of one or more of several types of low-trauma fractures or through the use of fracture risk algorithms such as FRAX. It has been suggested that either of these ways of predicting an increased fracture risk should also enable the use of the diagnostic term osteoporosis. It is the purpose of this paper to make the case that we should formalize this concept and encourage clinicians to use the term osteoporosis when they identify an older patient with an elevated fracture risk determined by any one of these criteria. [27]

The diagnosis of osteoporosis should be made on the basis of risk or prior fragility fracture. With this approach, the terminology and treatment of osteoporosis succumbs to the notion of risk; if the patient is at elevated risk, then the patient should be treated. We have returned to the definition of osteoporosis as originally defined by the T-score, but now, it encompasses the totality of risk, not just the T-score.” [29].

Here, osteoporosis is not low bone mineral density. It is having a fragile skeleton. It is being at high risk of sustaining a fragility fracture.

### Fracture risk as a disease

Some may argue that the use of risk predicting tools such as FRAX is of little philosophical significance to conversations about concepts of disease. Peter Schwartz has argued that such “risk-based diseases” are not actually diseases, and that treating them as such reflects “an unfortunate trend towards reclassifying risk as disease” [44]. Boorse considered such risk-based conditions as instances of instrumental health, and not as instances of disease [1]. Boorse argues that risk factors and diseases are “badly confused” by clinicians today [2]. Even so, there are good reasons to accept risk-based conceptions of osteoporosis as a proper disease, as many clinicians suggest.

Firstly, as Giroux has argued [7], it is sometimes difficult to distinguish risk factors and functional parameters. Arguably osteoporosis—as defined by the WHO in 2007—does involve a reduction in the function of bone, as assessed using bone mineral density. As FRAX makes use of bone mineral density measurements, it is not simple to dismiss this concept of osteoporosis as a purely risk-based condition.

Secondly, Giroux has argued that epidemiology itself can contribute to the measurement of functional efficiency [7]. She argues that simple measurements of physiological parameters do not necessarily constitute the measurement of functional efficiency. So, with respect to osteoporosis, bone strength might be the function of bone, and not bone mineral density. Bone mineral density is, on this view, just an indicator of strength or fragility. As Giroux suggests, epidemiology is used to improve the assessment of fragility provided by bone mineral density measurements by assessing the risk of fracture. Arguably, risk is being used here to assess functioning.

Thirdly, function-based and risk-based conceptions of osteoporosis are used to achieve the same medical goals. Schwartz argues that risk-based diseases (such as osteoporosis) are not actually diseases, because they do not have dysfunction [44]. This argument assumes a dysfunction requiring account of disease (such as Boorse’s), but does not try to justify this assumption. Schwartz does argue that the distinction between function-based conditions (diseases) and risk-based conditions should be maintained, because he wants to maintain the distinction between treating disease and assessing risks. Schwartz sees these as two distinct medical projects: curative medicine (my terminology, not Schwartz’s) that treats disease, and preventive medicine that prevents disease. Confusing these projects might lead patients to think they have a function-based condition (a disease) that really needs treating, as opposed to some degree of risk of disease developing, which might not [44, 45].

This attitude influences philosophers’ intuitions about the relevance of risk-based conditions to the debate about concepts of disease. As preventive medicine seeks to prevent disease, the conditions it addresses cannot themselves be diseases. Only curative medicine, which addresses function-based conditions, is seen as addressing proper diseases.

What this attitude misses is that preventive medicine does not (necessarily) focus on the prevention of disease. Rather, preventive medicine seeks to prevent *symptoms* of disease, and the *suffering* of patients. Assessing function is one of the main tools preventive medicine uses to do this, by detecting asymptomatic dysfunction, and addressing it before symptoms develop. The problem with this approach is that many patients with some level of functional efficiency will never develop symptoms, whereas others will. Consequently, some level of functional efficiency does not indicate that patients will develop symptoms, only that they have a certain risk of doing so. Even if a patient has reduced functional efficiency, they may not need treatment, as the risk of developing symptoms is low. Focusing on function-based conditions can encourage overtreatment (treating patients who would not have developed symptoms anyway), just as focusing on risk-based conditions can. So maintaining the distinction between function-based and risk-based conditions does not necessarily address the problem of overtreatment. Indeed, Schwartz [46] has argued that risk of ‘significant negative consequences’ (e.g. symptoms) should be considered when defining dysfunction. Curative medicine, which addresses function-based conditions, and preventive medicine, which only addresses risk-based conditions, are not distinct projects. Therefore, risk-based, preventive medicine is a proper domain to explore when investigating concepts of disease.

Doctors addressing asymptomatic dysfunction and doctors addressing risk-based diseases are both engaged in the same project: the prevention of symptomatic dysfunction. Bone mineral density measurements were chosen to define osteoporosis precisely because doctors hoped they could be used to predict (and thus prevent) fragility fractures and symptoms. FRAX was developed to improve this predictive ability, and not as part of a completely different medical project. Many of the patients diagnosed with osteoporosis using FRAX are the same as those diagnosed with low bone mineral density alone. Why should they be considered as merely having a risk-based condition, and not a proper disease, just because clinicians have decided to address the problem of whether they will develop symptoms using a risk-based approach, rather than a recognizably function-based approach? Even if bone mineral density is understood as a measure of the function of bone, and a diagnosis made using FRAX as a risk-based condition, osteoporosis is not any less of a disease because it has shifted from a function-based to a more risk-based condition.

### Reference classes in FRAX: T-score and population adjusted reference classes

FRAX is designed to be used in two stages. In Europe (in contrast to the USA) there is no universal screening program for osteoporosis. Rather, new cases are identified on an “opportunistic case-finding strategy” [43]. Information about risk factors obtainable by clinical history and examination (body mass index, history of previous fracture, etc.), and used as an input for FRAX. The patient’s risk of fracture is evaluated on that basis, without measuring bone mineral density [41, 43]. The UK’s National Osteoporosis Guidelines Group (NOGG) recommends that should a patient’s risk of fracture be sufficiently high in this first stage, they can be started on treatment even without bone mineral density assessment [12]. If a patient’s risk of fracture is sufficiently low, bone mineral density testing will not be recommended, and the patient will not be started on pharmaceutical treatment. Patient’s with middling risks of fracture are referred for bone mineral testing, and a new risk of fracture is calculated taking this information into account [41]. Exploring how FRAX uses reference classes in this second stage, where the full battery of risk factors are incorporated into the model, is revealing of how reference classes are used in contemporary medicine.

FRAX makes use of a patient’s T-score, calculated using bone mineral density assessed at the femoral neck, and using a young female reference class. The database it uses, the NHANES database, is always the same [28]. The reference class is not adjusted to specific populations. However, the reference class here is not used to decide whether a contribution to survival or reproduction is typical and therefore a function. Neither is it used to decide whether a level of functioning typical and therefore healthy. The reference class has a different function to that described by the BST: it is used to generate a risk factor that FRAX incorporates into its model of fracture risk.

However, populations are important for FRAX. Each of these risk factors carry a different significance to the model. Hazard ratios for each risk factor are calculated, which are the ratio fracture rates amongst people with the risk factors compared to those without the risk factor, all other things being equal. The degree to which the hazard ratios are independent of other risk factors is also assessed. On these grounds, risk factors become “successful,” “moderate,” or “weak” [47]. FRAX also takes account of death as a hazard that would compete with fracture, which influences the weighting given to risk factors. For example, when the ratio of fractures amongst smokers and non-smokers is compared, current smoking is a moderate risk factor for fracture. Even though smoking therefore makes a patient more likely to sustain a fragility fracture, it also makes the person more likely to die, which means they might die before they sustain a fragility fracture. This competing hazard is taken into account by FRAX. With death recognized as a competing hazard, smoking becomes a weak risk factor for ten-year fracture risk, as smoking also increased the risk of death [47]. The weight each risk factor is given by FRAX thus depends upon the specific population on which the model was trained and tested. Different populations with different epidemiological characteristics have different ‘strengths’ assigned to different risk factors. Indeed, FRAX is calibrated according to different national populations. FRAX uses different models for the population of the UK than it does for the population of Argentina [47]. Consequently, the presence of the same risk factors in two people with the same bone mineral density can result in these people having different risks of fracture, depending upon the national populations from which these people are drawn. The weighting of risk factors is population sensitive.

This finding challenges Boorse’s assertion that epidemiologists never “claim that the same condition (e.g., diastolic blood pressure over 90) is a disease in one group but not in another group” [2]. If disease status is determined by reaching a certain risk of fracture (as it sometimes is, see below), then people with the same bone mineral density (and other risk factors) may have different risks of fracture, depending on whether they are part of the UK or the Argentinian population. If bone mineral density and other risk factors define the condition a patient has, and risk of fracture defines disease status, then the relationship between condition and disease status can vary with epidemiological population.

It is debatable whether this makes these populations reference classes. An individual’s risk of fracture is not being compared to the average for that population. These populations are not being used to determine whether an individual is typical for that population. Nevertheless, a risk factor’s standard contribution to the risk of fracture is determined within that specific population, just as an organ’s standard contribution to survival or reproduction is determined within a specific reference class in the BST. These risk factors can only be used to calculate an individual’s risk of fracture relative to that specific population. It is impossible to determine this risk unless the population to which that individual belongs is specified using this approach, just as it is impossible to determine what the functions of an individual’s organs are without specifying the reference class to which that individual belongs using the BST. There are good grounds for considering these populations to be reference classes, even though they have a different role to that imagined by the BST.

### Converting risk into interventions: fixed and age-dependent thresholds

Once an individual’s ten-year risk of fracture has been calculated, this value still needs translating into disease status^8^, just as levels of functioning need translating into disease status by determining whether they are typical in the BST. Different countries do this in different ways, largely by either following the approach taken by the UK’s National Osteoporosis Guideline Group (NOGG), or by the USA’s National Osteoporosis Foundation (NOF) [47]. The NOF advocate “fixed” intervention thresholds, according to the risk of fracture. The particular level of risk they recommend as a treatment threshold is “based on a health economic assessment”, which is an evaluation the cost effectiveness of making interventions at different thresholds [47]^9^. Specifically, when a patient has a 20% chance of sustaining a major osteoporotic fracture (of the hip, forearm, humerus or spine [43]), the NOF recommend starting treatment, *regardless of the patient’s age or sex*. Here, all patients are grouped together in one large class, and those with the same risk of fracture get the same treatment [47, 48].

The NOGG are in some respects similar to the NOF. In both organizations, before they started to use FRAX, treatment had been recommended in patients who sustained a fragility fracture. The NOGG used this idea to set intervention thresholds for levels of risk calculated by FRAX. As a postmenopausal woman with a fragility fracture would be eligible for treatment, even if she had no other clinical risk factors and even if her bone mineral density was unknown, the NOGG reasoned that any person with the same level of risk of fracture should also receive treatment [43, 49].

Briefly, the NOGG guidance ‘translated’ the preceding Royal College of Physicians guideline which indicated that women with a prior fragility fracture may be considered for intervention without the necessity for a BMD test for the purpose of making the treatment decision. The translational logic used is that if a woman with a prior fragility fracture is eligible for treatment, then a woman with the same fracture probability but, in the absence of a previous fracture, should also be eligible. [48]

However, in contrast to the NOF, the NOGG did not set this threshold for treatable risk of fracture at one level for all patients. They set the trigger for treatment for women without a fragility fracture at the same level of risk as that for women with fragility fractures *of the same age.* This is an age-dependent threshold [49]. This threshold rises with age, because the risk of fracture in all people rises with age, including those people who have sustained a fragility fracture. So, the risk of sustaining a major osteoporotic fracture (at the hip, forearm, humerus or spine) required to qualify for treatment at the age of fifty is seven per cent [43], but at the age of ninety is 34% [47]. The NOGG also checked whether their proposed thresholds were cost effective given the likely treatment patients with osteoporosis would receive.

So, the NOGG use age-adjusted reference classes, as the BST advises. However, NOGG do not use reference classes to define the average person. Their thresholds were set according to the level of risk experienced by people who have a fragility fracture, by people *with a disease.* Here it is similarities with diseased people, not dissimilarities with the average person, that establishes the presence of disease. Like the fracture threshold concept discussed above, the comparison here is with a *pathological reference class*: “In other words, the intervention threshold is set at the age-dependent ‘fracture threshold’” [48].

Even though the developers of FRAX provide separate table of intervention thresholds for men and women [43], the NOGG [12] use the same risk thresholds for men as they do for women, on the basis that the cost-effectiveness of treatment is the same for people at the same level of risk regardless of sex: “Note that the same intervention threshold is applied to men as in women, since the effectiveness and cost-effectiveness of intervention in men are broadly similar to that in women for equivalent risk” [48]. The treatment threshold is not set according to someone who has sustained a fragility fracture of the same age and sex. Rather, the level of risk deemed appropriate to start a man on treatment is set by comparison to women of the same age who have fractured.

Furthermore, this comparison is not drawn between the person whose fracture risk is being evaluated and women from all over the world. It is drawn between that person and *other women from the same country*. Just as the ‘strength’ of different risk factors varies between epidemiological populations, the overall risk of fracture for women with any combination of risk factors also varies between epidemiological populations. Consequently, the reference class used to judge whether an individual’s risk of fracture is acceptable *for their society* is allowed to vary with national population.

The intervention threshold will vary from country to country because the population risks (of fracture and death) vary. The fracture probability in women with a prior fracture in the five major EU countries is shown in Fig. 9. Probabilities are highest in the UK and lowest in Spain. [48]

Although the NOGG [12] do distinguish between reaching a risk of fracture as assessed by FRAX that constitutes a therapeutic threshold and the diagnosis of osteoporosis, please recall that other clinicians do not. As this threshold is higher in the UK than in Spain, there is a level of risk which is considered a disease (by some) in Spain, but not in the UK. Against Boorse, this is disease in the Spanish male, but not in the UK male. Epidemiological interest in different populations generates the view that the same condition (a particular risk of fracture) is a disease in one population but not in another.

Here, then, there is another use of a reference class. To turn fracture risk into a treatment threshold (and, to some, a diagnostic threshold) for any person, the NOGG use an age-adjusted, female, pathological, and national population specific reference class; to diagnose osteoporosis in all adults, including men [43, 49].

It is possible to get an idea about how clinicians choose between “fixed” and “age-depended” approaches to setting intervention thresholds by looking at research that tries to combine the approaches, to produce a “hybrid model” [48]. Researchers in Lebanon have suggested a model that uses a fixed threshold until patients reach the age of seventy, and age dependent thresholds thereafter. Researchers in the UK have done the reverse, by suggesting a model that uses age-adjusted thresholds until patients reach the age of seventy, and fixed thresholds thereafter.

In the UK, the NOGG have recently updated their guidance to fix the intervention threshold after seventy years of age [12]. They did this to correct discrepancies between estimates of fracture risk made on the basis of clinical risk factors alone and those made including bone mineral density testing as well. It is commonplace for patients to receive treatment on the basis of having a fragility fracture without having bone mineral density measured. Should the bone mineral density of these patients be measured, it is possible for this new information to reduce their risk of further fracture, and reduce it to below the level of other patients who are not receiving treatment [48]. They argue that this situation becomes more common in older patients. By capping the risk of fracture needed to start treatment at 20% in patients aged seventy and above, the NOGG hope to capture most of these other patients without capturing too many people who will never fracture [48]. Lowering the intervention threshold in the elderly by capping it at 20% also has the effect of reducing the need of bone mineral testing in this population, which is both logistically difficult and costly [48].

In Lebanon, adopting a NOGG-like model, where treatment is started in patients with the same risk of fracture as a woman of the same age with a fragility fracture, is seen as suboptimal. This is because it would lead to many women being treated without having a fracture and with only a ten year-risk of fracture 10% or below. Clinicians decided that this would constitute overtreatment, and would not be an optimal way to allocate health resources [48]. Instead, a fixed treatment threshold is set at 10% until the age of seventy, after which it is allowed to rise was allowed to rise with age, as there are a great many elderly people with a ten-year fracture risk of more than 10%.

Choosing reference classes to set diagnostic and intervention thresholds using FRAX is done differently in different countries. This choice is made according to a complex mixture of often competing intuitions and ideas. These include the views that patients with the same level of risk should receive the same level of care; that the risk of fracture rises with age should be taken into account; that differences between national populations should be taken into account; that as many of the patients who are going to fracture and who will benefit from treatment should be captured as possible; that the patients captured who will not fracture and who will not benefit from treatment should be as few as possible; that management of patients should be cost-effective, and fit within the healthcare priorities of different nations.

Exploring how FRAX is used to assess risk of fracture, diagnose disease and direct treatment reveals the use of many different kinds of reference classes at several places in the model. Firstly, FRAX uses a young female reference class to calculate a T-score for both men and women. Secondly, it selects and adjusts the ‘strength’ of risk factors according to the characteristics of specific epidemiological populations. Thirdly, it translates risk of fracture into an intervention threshold, or even, to some, a diagnostic threshold. In doing this, those who use FRAX mix and match many different sorts of age and population relative reference classes. The choice of which sort of reference class to employ cannot be reduced to the outcome of debates about which is the correct natural class of organisms. Rather, these are accompanied by a plethora of more pragmatic considerations that often overwhelm them.

## Conclusion

At first glance, osteoporosis is philosophically interesting because clinicians use a young reference class to define it in older women, using the T-score. Further examination revealed that the choice of reference class is even stranger than this, as a young female reference class is also used to define the disease in older men. Even though clinicians do value the intuition that patients should be compared with a normal population of patients of the same age and sex, this intuition is subordinated to others. The intuitions that patients with a high risk of fracture should be treated, regardless of their age, and that patients with the same risk of fracture should receive the same diagnosis, regardless of their sex, take precedence in this case. Even using *pathological* reference classes is an option, in which an individual is compared to a diseased population instead of a normal population. The choice of reference class is not determined by clinicians’ views about which groups are natural classes of organisms with uniform functional design.

Intuitions about the importance of fracture risk are so powerful that some clinicians argue for osteoporosis to be defined as having a high risk of sustaining a fragility fracture, using tools like FRAX, even if this risk is evaluated using factors in addition to the measurement of bone quantity or quality. I have argued that this is not unreasonable, because tools like FRAX incorporate measures of bone quantity; because fragility is arguably the proper measure of dysfunction, and this can be assessed in terms of risk; and because the diagnosis of osteoporosis is made to help prevent fragility fractures, whether this is understood as a function-based or as a risk-based condition. In this case, epidemiology does play an important role in determining how fragile a person’s skeleton is, and in determining the degree of fragility considered diseased. Against Boorse, the same condition can be a disease in one population but not in another.

In contrast to the assumptions expressed in the BST, risk-based tools like FRAX use several reference classes at once. FRAX uses the young female reference class to generate the T-score, and then a population-based reference class to assess the strength of its risk factors. Organizations such as the NOGG and the NOF take the results of FRAX and use yet more reference classes to set an intervention threshold. Until recently, the NOGG used a population specific, female, age-adjusted and pathological reference class to set a threshold for intervention at the same level of risk as a women of the same age, from the same national population, who has sustained a fragility fracture. Other organizations such as the NOF set a fixed threshold for intervention, using an age and sex unadjusted reference class. Today, the NOGG use a hybrid reference class, adjusting for age until seventy years, and then setting a fixed threshold after that. This shows that clinicians do have to make choices about how to use reference classes to define disease. They make these choices by considering effectiveness and cost effectiveness of diagnostic and therapeutic interventions. The ethical intuition that patients with the same level of risk of fracture should receive the same level of care plays a central role in the construction of reference classes. This is a reasonable foundation for concepts of health and disease, but it is different to that proposed by the BST. Clinicians find the above factors are more important to the choice of reference class than is the intuition that patients should be compared with others of the same natural class of organisms with uniform functional design. In this light, the choice of reference class, and thus the choice of disease concept, looks much less naturalistic and much more pragmatic.

## Data Availability

N/A.

## Footnotes

2 Boorse notes in various places that race might also be relevant to defining reference classes (e.g., [1, p. 558]).

3 Many medical professionals use reference classes, including clinicians, epidemiologists and medical researchers. I use the term ‘clinician’ to refer to all such medical professionals.

4 Here Boorse refers to the Framingham Heart Study, which is widely held to be the origination of the risk-factor approach to medicine [8–10].

5 The WHO (1994) [17] cite Clarita Odvina et al. [31] as researchers who advocate this approach. Odvina et al. [31] do discuss setting the fracture threshold at 2 standard deviations above the fracturing populations mean, but also discuss other methods of setting a fracture threshold. They use several different logistic regression techniques to try to derive this threshold as the bone mineral density where the probability of fracturing falls to zero, the bone mineral density where the number of fractures sustained by patients falls to zero, and the point where there is a substantial decrease in the probability of fracturing. Each of these approaches represent subtly different concepts of disease.

6 Other medical researchers have suggested using the rate of bone mineral density decrease, instead of its absolute level, to detect patients most likely to fracture [33–35]. This brings to mind yet another concept of disease—the possibility of comparing an individual’s bone mineral density to that of their younger self, as opposed to the average for a population—without actually putting this concept into practice.

7 This indicates that diagnostic categories are sometimes designed according to the treatment options available, as several authors have argued [36–39]. It is beyond the scope of this paper to explore the influence of treatment options on disease concepts, but this is nonetheless worth noting.

8 These clinicians use diagnostic thresholds as treatment thresholds. Again, this raises issues about the connection between treatment options and disease status.

9 Some countries adopt a different threshold for intervention. For example, Japan and Sweden use a 15% ten-year risk of major osteoporotic fracture as an intervention threshold [47]. So, the fixed threshold approach is population dependent in this sense.

## Code availability

N/A.
